# High-Accuracy Recognition Method for Diseased Chicken Feces Based on Image and Text Information Fusion

**DOI:** 10.3390/ani15152158

**Published:** 2025-07-22

**Authors:** Duanli Yang, Zishang Tian, Jianzhong Xi, Hui Chen, Erdong Sun, Lianzeng Wang

**Affiliations:** 1 College of Information Science and Technology, Hebei Agricultural University, Baoding 071001, China; tzs010220@163.com; 2 Hebei Key Laboratory of Agricultural Big Data, Baoding 071001, China; 3 Graduate School, Hebei Agricultural University, Baoding 071001, China; xijianzhong@hebau.edu.cn; 4 College of Animal Science and Technology, Hebei Agricultural University, Baoding 071001, China; 5 Key Laboratory of Broiler and Layer Facilities Engineering, Ministry of Agriculture and Rural Affairs, Baoding 071001, China; 6 Hebei Taomu Geda Agricultural Science and Technology Co., Baoding 074300, China; lyxlwc@126.com; 7 Hebei Layer Industry Technology Research Institute, Handan 056007, China; 15030424480@163.com

**Keywords:** multimodal, ResNet50, BERT, chicken disease, cross-attention

## Abstract

Poultry feces, a critical biomarker for health assessment, requires timely and accurate pathological identification for food safety. Conventional visual-only methods face limitations due to environmental sensitivity and high visual similarity among feces from different diseases. To address this, we propose MMCD (Multimodal Chicken-feces Diagnosis), a ResNet50-based multimodal fusion model leveraging semantic complementarity between images and descriptive text to enhance diagnostic precision. Key innovations include the following: (1) Integrating MASA(Manhattan self-attention)and DSconv (Depthwise Separable convolution) into the backbone network to mitigate feature confusion. (2) Utilizing a pre-trained BERT to extract textual semantic features, reducing annotation dependency and cost. (3) Designing a lightweight Gated Cross-Attention (GCA) module for dynamic multimodal fusion, achieving a 41% parameter reduction versus cross-modal transformers. Experiments demonstrate that MMCD significantly outperforms single-modal baselines in Accuracy (+8.69%), Recall (+8.72%), Precision (+8.67%), and F1 score (+8.72%). It surpasses simple feature concatenation by 2.51–2.82% and reduces parameters by 7.5M and computations by 1.62 GFLOPs versus the base ResNet50. This work validates multimodal fusion’s efficacy in pathological fecal detection, providing a theoretical and technical foundation for agricultural health monitoring systems.

## Simple Summary

The health of chickens can be judged by observing their feces. Traditional methods rely on manual checks or image-only analysis, which can be inaccurate due to lighting or image quality issues. In this study, we propose a new method that combines images with text descriptions to better identify whether a chicken is sick. The system can both “see” the image and “understand” the written description, making the judgment more complete. Test results show that this approach is more reliable than using images alone, and it performs well even in complex farm environments. It helps farmers detect problems earlier, reduce medication use, and improve overall farming efficiency.

## 1. Introduction

Chickens are a major source of meat and eggs worldwide [1,2], and their health directly affects food safety and quality. Currently, the livestock industry relies on drugs to control more than 80 types of diseases in laying hens [3], but the health risks caused by drug residues have raised serious concerns. Therefore, the development of timely and accurate disease detection technologies for chickens has become a critical need to reduce drug use and prevent the spread of diseases.

Currently, the most common method for diagnosing sick chickens is manual observation, but this method has drawbacks such as strong subjectivity and high risks of zoonotic diseases. With the development of information technology, automated diagnostic methods for diseased chickens based on machine vision [4,5] have made significant progress (e.g., infrared thermal imaging [6], comb contour analysis [7], foot feature classification [8], and Lab color space detection [9]). However, their reliance on manually designed features limits their generalization capabilities. In recent years, deep learning technology [10,11] has driven animal and plant pathology detection into a new phase: Zhou et al. [12] used Faster R-CNN to detect abnormal chicken feces, while Mizanu et al. [13] utilized YOLO-V3 to segment regions of interest (ROI) from feces images and employed ResNet50 for disease classification of the segmented images. Chen et al. [14] used YOLOv5s and tracking technology for the early detection of respiratory diseases in chickens, while Thakur [15] proposed a Transformer-based automatic disease detection model “PlantViT” for identifying plant diseases.

Chicken manure serves as a non-invasive, sensitive key biological indicator. Abnormal changes in its visual features can provide direct pathological evidence for early warning of diseased chickens. However, current single-modal representations in deep learning struggle to fully capture the complex pathological features of chicken manure, particularly facing the following three challenges: (1) Weak feature expression: Visual features of feces during the incubation period are not prominent, and there are differences in feces features among individual diseased chickens, leading to blurred feature boundaries. (2) Modal limitations: Single-modal images/text lack the ability to comprehensively characterize pathology, resulting in models unable to extract sufficiently specific and robust features from a single modality. (3) Environmental interference: Factors such as litter color and uneven lighting reduce model robustness.

Multi-source data fusion is emerging as a new approach to overcome these challenges [16]. Dai et al. [17] used image–text fusion (ITF) to identify pests, while Wang Chunshan [18] et al. embedded disease text modality information on the basis of disease image modality, achieving joint feature representation learning. Lee et al. [19] combined multimodal data with hybrid data augmentation techniques to simultaneously predict crop type, detect diseases, and assess disease severity. Ma et al. [20] dynamically fused multispectral images (MultimodalNet) to predict crop yield, Chen Wenjun et al. [21] constructed a deep-near-infrared lightweight framework (YOLO-DNA), and Liu Yang et al. [22] integrated tomato image, near-infrared spectral, and tactile modality information through feature fusion. These studies confirm that strategies such as feature concatenation, weighted fusion, and GAN [23] can significantly improve model adaptability. However, existing methods still face the challenge of balancing information redundancy and computational efficiency.

Given the unique characteristics of testing sick chicken feces, this paper proposes a multimodal fusion model called MMCD, with core innovations including the following:

(1) Dual-modal feature complementarity: Fusing ResNet [10] visual features with BERT [24] text semantic features to enhance the model’s sensitivity to weak pathological features.
(2) Dynamic gating [25] fusion mechanism: Replacing feature concatenation with Gated Cross-Attention (GCA) to filter environmental noise and reinforce key pathological features.
(3) Lightweight architecture: Gated Cross-attention (GCA) reduces redundant computations, avoids overfitting of weakly correlated information, and improves fusion efficiency and training stability.
(4) Data augmentation strategy: Low-quality datasets are constructed using image degradation techniques to enhance robustness in complex scenarios.

Experiments demonstrate that MMCD significantly improves early pathological identification accuracy and generalization performance, providing a new paradigm for intelligent monitoring systems in livestock farms.

## 2. Materials and Methods

### 2.1. Data Sources

The chicken feces image dataset used in this study is from the Zenodo open database [26,27]. Built specifically for disease diagnosis scenarios for small and medium poultry farmers, the dataset was collected by the producer between September 2020 and February 2021 in the Arusha and Kilimanjaro regions of Tanzania. In the process of data collection, mobile phones were used to capture images with the Open Data Kit (ODK) application.

**Figure 1:**
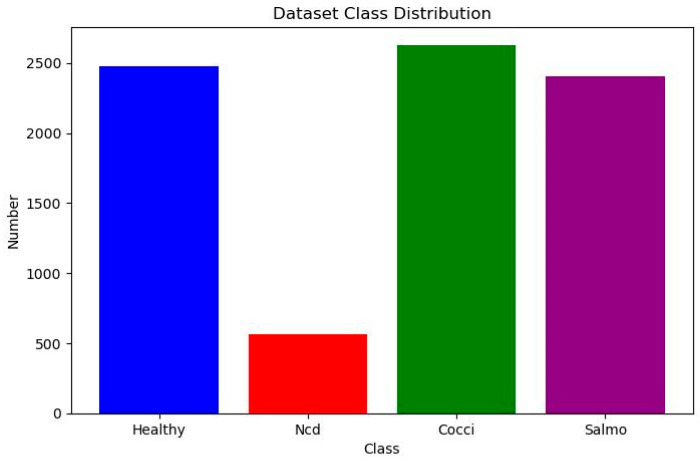
Dataset class distribution.

The original dataset contains a total of 8067 RGB three-channel images (size 224 × 224 pixels, image format JPG) covering four classes of labels: “cocci” (2476 images), “ncd” (562 images), “salmo” (2625 images) and “healthy” (2404 images). The label information of each image was managed through CSV files. The dataset category distribution is shown in Figure 1.

### 2.2. Data Preprocessing

**Figure 2:**
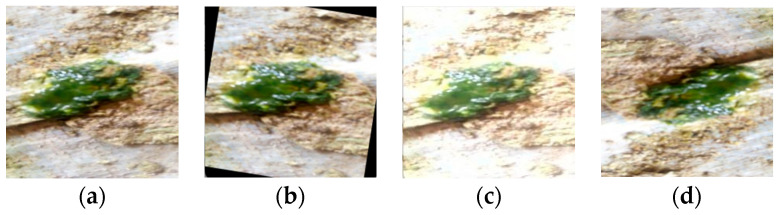
Data augmentation effect visualization; (**a**) original image; (**b**) rotate; (**c**) random brightness; (**d**) flipping.

Figure 1 depicts the data volume distribution across different chicken manure categories within the dataset. The figure reveals a significant imbalance in sample sizes among categories, with several exhibiting notably low data volumes. This imbalance can lead to substantial variations in recognition accuracy, particularly for under-represented categories. To address this issue and improve model generalization, data augmentation techniques—including rotation, random brightness adjustment, and flipping—were applied to expand the “ncd” category. Consequently, the number of “ncd” samples increased from 562 to 2300, resulting in a total dataset of approximately 9800 chicken manure images. The outcomes of this augmentation are presented in Figure 2.

**Figure 3:**
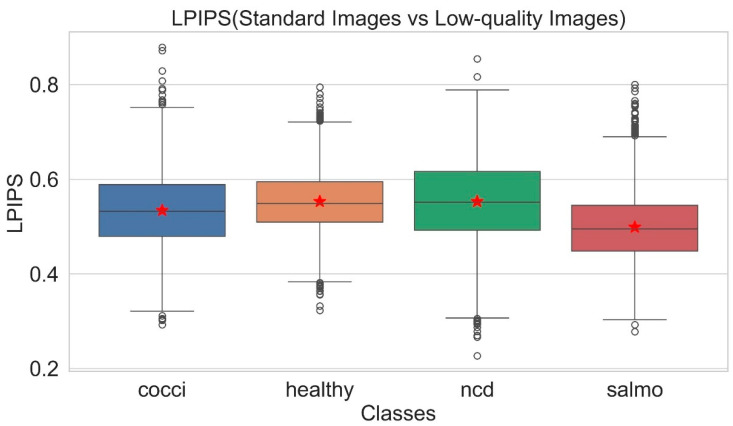
Boxplot of LPIPS scores.

To enhance the model’s robustness in complex environments and to develop challenging low-quality image datasets, this study implements three data degradation strategies based on the original image data: (1) Gaussian noise (σ = 20) is introduced to replicate random interference caused by device sensors or the transmission process during imaging. (2) Gaussian blur (σ = 2.0, k = 13 × 13) is applied to mimic the degradation of image sharpness resulting from motion blur or focal length deviation. (3) The brightness of the image (Δ = ±30) is adjusted by randomly increasing or decreasing the brightness value to simulate the impact of varying illumination conditions on imaging quality. These methods are designed to improve the model’s adaptability to complex imaging conditions encountered in real farm settings. The LPIPS score [28] serves as the evaluation metric to assess the extent of image quality degradation. Figure 3 presents box plots of LPIPS scores for both low-quality and standard images.

As can be seen in Figure 3, the median LPIPS (horizontal line in the box) of the four categories of images were all in the range of 0.4–0.6 (the stars are the mean values), indicating that the degraded images did not completely lose the original category distinguishing features of the fecal images, although there were some perceptual differences between the degraded images and the standard images.

In this study, we delineate the objectives and methodologies associated with “data augmentation” and “image degradation.” Data augmentation primarily addresses issues of class imbalance and overfitting during training, employing techniques such as geometric transformations and subtle color perturbations. Conversely, image degradation focuses on generating low-quality image datasets to enhance the model’s robustness against various forms of image degradation, including Gaussian blur, Gaussian noise, and significant brightness perturbations. These two approaches are distinctly operationalized to ensure the model’s capacity to adapt to both semantic diversity and quality variations concurrently.

Furthermore, to mitigate the influence of a singular textual description style on model training, four experts in the field of animal husbandry were enlisted to provide subjective descriptions of the appearance characteristics (including color, shape, and moisture content) of the feces of diseased chickens. The textual data obtained were then aligned with image samples and recorded in a CSV file to facilitate subsequent multimodal classification and recognition tasks. Some samples and their corresponding information are shown in Table 1.

**Figure 4:**
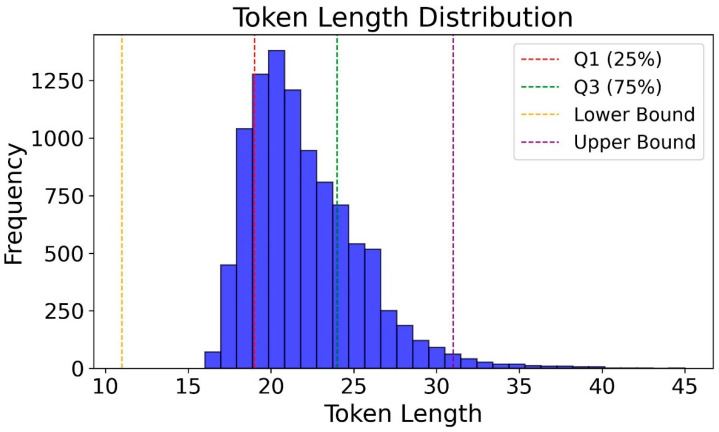
Token length distribution.

The boxplot of token length distribution for text data is shown in Figure 4, using BERT Tokenizer to automatically preprocess text. IQR index analysis shows that when the vectorized text length is set to 30 characters, the padding overhead can be reduced and the calculation cost can be reduced, while retaining most of the text information, so the text length is set to 30.

To ensure the effectiveness of the model’s training, the multimodal fusion dataset was partitioned into a training set, a validation set, and a test set in a ratio of 7:2:1, and the specific numbers are shown in Table 2.

### 2.3. Dual-Modality Fusion Model for Diagnosis of Diseased Chicken Feces

The image processing component employs the ResNet architecture to extract features, including color and texture, from images. In contrast to traditional convolutional networks, ResNet incorporates a residual structure that concatenates input features with extracted features, thereby facilitating the effective training of deep networks without performance degradation. Compared to the Transformer model, ResNet is more computationally efficient and demands fewer resources. The text processing component utilizes a pre-trained BERT network to extract semantic and contextual relationship features from the text. By integrating these two distinct modalities, the approach addresses the limitations inherent in single-feature representation and leverages the complementarity between different modalities to enhance the model’s detection performance.

**Figure 5:**
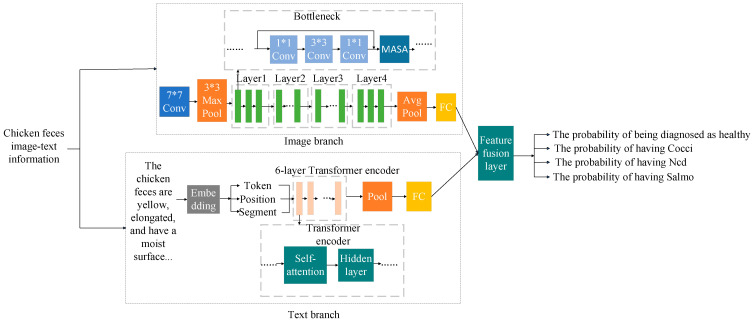
Dual-modal fusion model for diagnosing diseased chicken feces.

The structure of the model is depicted in Figure 5. The image processing network employs ResNet50, comprising four layers with residual blocks (Bottleneck) in quantities of 3, 4, 6, and 3, respectively. The dimension of the fully connected layer is modified to 768 to align with the output dimension of the text processing network. The text processing network utilizes a BERT model, which encodes input text through word embeddings, position embeddings, and segment embeddings. It then processes the text through 12 layers of Transformer encoders, with the number of fully connected layers adjusted to suit the subsequent classification task. The extracted image and text features are integrated within the feature fusion layer. The fusion methods employed include simple concatenation (Concat), a Transformer-based cross-modal encoder, and a Gated Cross-Attention (GCA). Ultimately, the model generates predicted probabilities for each category, which are utilized for model training and classification tasks.

#### 2.3.1. MASA Mechanism

Considering that low-quality images often suffer from blurring and significant noise, and that chicken manure is distributed in a scattered manner in some images, we incorporated the MASA module [29] into the image processing network to enhance feature extraction accuracy. This module integrates a channel attention mechanism with a spatial weighting mechanism based on the Manhattan distance, allowing for adaptive multi-scale information fusion. As a result, it effectively captures both local and global information within the input feature maps. By facilitating adaptive multi-scale fusion during feature extraction, the MASA module extends the network’s receptive field. Furthermore, through dynamic attention weighting, it enables the network to concentrate more precisely on critical features across different scales and spatial positions.

**Figure 6:**
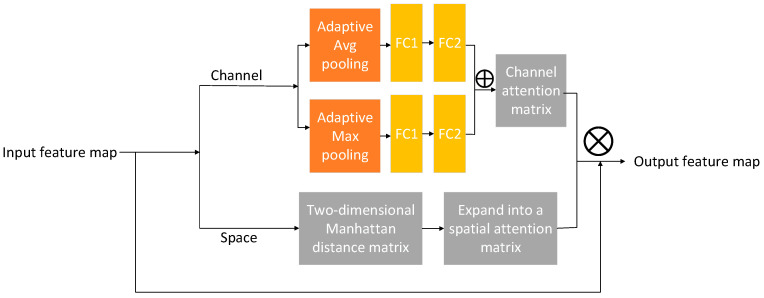
The structure of the MASA (Manhattan self-attention) module.

Following the maximum pooling operation, the feature maps are subsequently fed into the Bottleneck structure of the ResNet50 architecture. After undergoing convolution, these feature maps are directed to the Manhattan Attention (MASA) module, which is strategically positioned after the third 1 × 1 convolution layer of the Bottleneck. This placement is intended to optimize the effectiveness of the MASA mechanism in enhancing critical information following multi-scale feature extraction (refer to Figure 6 for the structural details of the MASA module). Within the MASA module, the feature maps are initially subjected to global pooling across the channel dimension, incorporating both adaptive average pooling and adaptive max pooling, which results in the generation of two independent feature maps. Subsequently, two fully connected layers execute nonlinear mapping operations to facilitate both dimensionality reduction and dimensionality expansion, thereby enabling the extraction of more refined features. Ultimately, the two channel attention matrices are combined to produce the final channel attention matrix. The formula for generating the channel attention matrix is expressed as (1).

(1) $$A_{channel}=\delta (W_{2}\left(\partial \left(W_{1}\cdot P_{1}\left(X\right)\right)\right))+W_{2}(\partial \left(W_{1}\cdot \left(P_{2}\left(X\right)\right)\right))$$

In the formula, *A_channel_* represents the channel attention matrix, *X* ∈ R^(B × C × H × W) is the input feature map, *P*_1_ is the adaptive average pooling layer, *P*_2_ is the adaptive max pooling layer, *W*_1_ and *W*_2_ are fully connected layers, *W*_1_ ∈ R^(C/r × C) is used to compress the number of channels, *W*_2_ ∈ R^(C × C/r) is used to restore the number of channels, ∂ is the ReLu activation function, δ is the Sigmoid function, and normalization is performed.

In the spatial dimension, two-dimensional grid coordinate matrices corresponding to the height and width are initially generated. Utilizing these 2D grid coordinates, the Manhattan distance between each pixel in the feature map is computed and subsequently normalized by dividing it by the sum of the height and width (H + W), as illustrated in Equation (2). This process results in the formation of an H × W Manhattan distance matrix, where H denotes the height and W denotes the width. To ensure compatibility with the input feature map, this matrix is expanded to match the full dimensions of the feature map, represented as B × C × H × W, where B signifies the batch size and C represents the number of channels.

(2) $$A_{spatial}=\frac{\left|i-m\right|+|j-n|}{H+W}$$

where (*i*,*j*) and (*m*,*n*) denote the position of two pixels in the feature map.

The resultant channel attention matrix is subjected to a dot product operation with the spatial attention matrix to derive the fusion matrix. Subsequently, the fusion matrix is element-wise multiplied with the original feature map to produce the final feature map, as illustrated in Equation (3).

(3) $$X_{out}=X⊙(A_{channel}⊙A_{spatial})$$

where ⊙ stands for element-dot multiplication, X represents the original input feature map, and $X_{out}$ represents the output feature map. $A_{channel}$ represents the channel attention matrix, and $A_{spatial}$ represents the spatial attention matrix.

#### 2.3.2. Depthwise Separable Convolution

**Figure 7:**
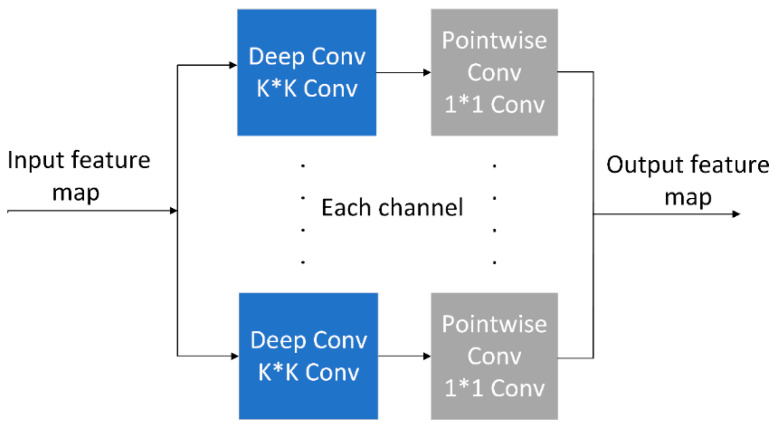
The structure of Depthwise Separable convolution.

After adding the Masa module, the ResNet50 network contains more standard convolutional layers, and the structure is bulkier. In order to appropriately reduce the amount of parameters and calculation, the convolution in the model is replaced by the Depthwise Separable convolution (DSconv). Compared with traditional convolution, DSconv [30] is composed of Depthwise convolution and pointwise convolution. Firstly, Depthwise convolution is performed on each input channel separately, and then Pointwise convolution is used to fuse channel information and output to each new channel. The linear combination between the channels is realized, and the output feature map is finally obtained. The Depthwise Separable convolution structure is shown in Figure 7. The traditional convolution calculation formula is (4), and the Depthwise Separable convolution calculation formula is (5).

(4) $$Y\left(i,j,o\right)=\sum_{c=0}^{C_{m}-1}\sum_{m=0}^{K-1}\sum_{n=0}^{K-1}X(i+m,j+n,c)\cdot W(m,n,c,o)$$

In Equation (4), Y(i,j,o) represents the value of the o-th channel of the output feature map at the position (i,j). Meanwhile, X(i+m,j+n,c) denotes the value of the c-th channel of the input feature map at the position c(i+m,j+n). Additionally, W(m,n,c,o) signifies the weight of the convolution kernel corresponding to the c-th input channel and the o-th output channel.

(5) $$Y\left(i,j,o\right)=\sum_{c=o}^{C_{m}-1}(\sum_{m=0}^{K-1}\sum_{n=0}^{K-1}X(i+m,j+n,c)\cdot W_{depth}(m,n,c))\cdot W_{point}\left(c,o\right)$$

In Equation (5), $X\left(i+m,j+n,c\right)$ is the pixel value of the input feature map at position $\left(i+m,j+n\right)$ and the c-th channel, $W_{depth}\left(m,n,c\right)$ is the convolution and weight of the K×K depth convolution kernel at the c-th channel, and $W_{point}\left(c,o\right)$ is the weight of the 1×1 pointwise convolution kernel.

The decomposition of standard convolution into deep convolution, which utilizes single-channel convolution operations, and pointwise convolution, which employs 1 × 1 convolution operations, markedly reduces the number of convolution parameters and computational complexity, while preserving the robust feature representation capabilities inherent in convolutional processes.

#### 2.3.3. Text Processing Network

**Figure 8:**
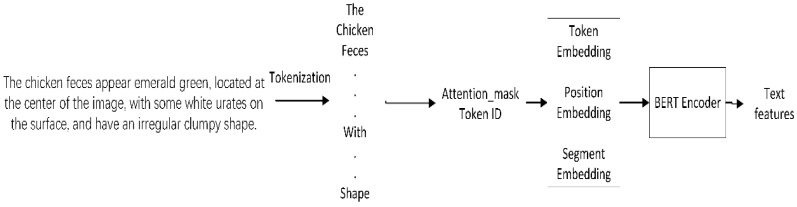
Text feature extraction process.

When the BERT model is used to extract text features, the input text is first fed into the WordPiece tokenizer to split the sentence into sub-word units. After word segmentation, the tokens are mapped to BERT vocabulary index IDs, and an attention_mask is generated, which is used to indicate the actual parts of the text the model should pay attention to. As shown in Figure 8, the generated formula of token ID and attention_mask is shown in Equation (6). Then, the token ID and attention_mask are fed into BERT model.

(6) $$Token\;IDs=\left\{t_{1},t_{2},t_{3},\ldots \ldots ,t_{n}\right\}\in N^{n}\;attention\_mask=\left\{\begin{array}{c} 1\\ 0 \end{array}\right.$$

where $t_{n}$ denotes the index in this table for the nth token; $attention_{mask}=1$ denotes that token is a valid input and $attention_{mask}=0$ denotes that token is a padding fill.

BERT first encodes the input using Token Embedding, Position Embedding, and Segment Embedding to gain a context-dependent vector representation for each token. These vectors are then fed into multi-layer Transformers for deep semantic modeling. The encoding and deep semantic modeling formulas are shown in Equation (7).

(7) $$E_{i}=T_{i}+P_{i}+S_{i}\;H^{l}=FFN(MultiHead\left(H^{\left(l-1\right)}\right))$$

where $E_{i}$ represents the final input embedding of the ith token; $T_{i}$ represents the word vector of the corresponding token ID; $P_{i}$ represents the position information of the token in the sequence; $S_{i}$ used to distinguish sentence A/B, or 0 for a single sentence. l indicates the number of layers of the Transformer; $H^{\left(l-1\right)}\in R^{n*d}$ is the input to layers l-1, n is the number of tokens, and d = 768; MultiHead(*) is the multi-head self-attention mechanism; FFN(*) is a feedforward network; $H^{l}\in R^{n*d}$ is the output and is a contextual representation of each token.

Finally, the vector corresponding to the [CLS] token in the BERT output is extracted as a global semantic representation of the entire text, which is used as text features for subsequent tasks.

#### 2.3.4. Dual-Modal Feature Fusion Layer

**Figure 9:**
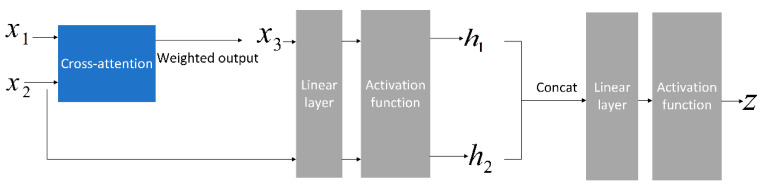
Gated mechanism structure diagram.

Concat is used to concatenate the extracted image and text features in the feature dimension, and then the combined features are input into the gating mechanism layer. The gating mechanism is used to filter and use the features, and the noise influence of irrelevant features on the recognition accuracy of the model is eliminated, as shown in Figure 9.

The operation process of the gated mechanism is shown in Equation (8). Where $x_{1}$ is the image feature and $x_{2}$ is the text feature; $h_{1}$ is the fusion feature after the cross-attention mechanism; $h_{2}$ is the linear transformation of text features and is the independent feature; z is the generated gated signal. After generating the gated signal, it is used to adjust the contributions of the two modes. The calculation process is as follows:

(8) $$z=\sigma (W_{1}x_{1}+W_{2}x_{2}+b)\;output=z\cdot h_{1}+(1-z)\cdot h_{2}$$

where σ is the activation function; $W_{1}$ and $W_{2}$ are the weight matrices, and both are learnable parameters; b is the bias term; and output is the final output. Where z≈1, the final output is biased towards the fused features $h_{1}$ and vice versa towards the independent features $h_{2}$.

## 3. Experimental Results and Analysis

### 3.1. Experimental Parameter Setting

The models involved in this study were all run on the same equipment, and the specific configuration is shown in Table 3. Image feature extraction used the ResNet50 network, text feature extraction used the BERT model, the length of input text (pad_size) was set to 30, the size of input image was set to 224 pixels × 224 pixels, the number of iterations (Epoch) was set to 100, and the training Batch size (Batch Size) was set to 16. The output feature dimension of the two modes was set to 768. The hyperparameter settings in the experiment were as follows: the initial learning rate was set to 0.00001, the cosine annealing algorithm was used for learning rate attenuation, Adam was selected as the optimizer, and He was used for initialization.

Accuracy, Recall, Precision and the harmonic mean of Precision and Recall (F1 score) were used as evaluation indexes. Accuracy is the proportion of samples predicted correctly by the model over all samples, which reflects the overall predictive ability of the model. Recall measures the proportion of samples predicted correctly as positive class, and Precision measures the proportion of samples predicted as positive class that are really positive class. The harmonic mean F1 score is used to measure the combined performance of the model’s Precision and Recall.

### 3.2. Comparative Experiments

To address the challenge of low classification accuracy when relying solely on image modalities for detecting diseased chicken feces, which is attributed to limited feature availability, this section introduces a multi-modal feature fusion strategy. Specifically, three fusion methods are employed: simple concatenation (denoted as ResBERT-C in the table), a Transformer encoder [31] (denoted as ResBERT-T in the table), and the novel model proposed in this study (MMCD). Furthermore, to assess the effectiveness of the fusion strategies, these methods are compared against a baseline model utilizing only the image modality (denoted as ResNet50 in the table) and its enhanced version (denoted as ResNet50-MD in the table).

As illustrated in Table 4, the ResNet50 model, utilized as the foundational image processing model, attained an Accuracy rate of merely 85.58%, the lowest among all evaluated models. This outcome suggests that exclusive dependence on visual features is inadequate for the precise identification of diseased chicken feces, particularly under conditions characterized by low image quality, high noise levels, and minimal inter-class variability. In contrast, the ResNet50-MD model, which integrates a multi-scale feature extraction mechanism, achieved an improved Accuracy rate of 89.24%, with Precision and Recall metrics enhancing to 89.44% and 89.24%, respectively, under the premise of lower parameter number (18.06 M) and smaller computational complexity (2.51 G). These findings substantiate the efficacy of the multi-scale design in capturing local image information, especially in modeling complex texture structures.

Compared with the single-modal model, the overall performance of the dual-modal fusion model is significantly improved. The ResBERT-C model effectively makes up for the lack of image information by introducing text semantic description. Its Accuracy is improved to 95.21%, the F1 value reaches 95.15%, while the parameter number is 60.71 M, and the computational complexity is 3.78 GFLOPs, showing a good balance between performance and efficiency. Although the ResBERT-T model further expands the depth of text modeling, the parameter number is greatly increased to 145.79 M, and the computational complexity is also 4.86 G, its Accuracy (95.11%) and F1 value (95.10%) are not significantly better than ResBERT-C, indicating that in the case of a significant increase in model complexity, the Resbert-T model has a good balance between performance and efficiency. The performance improvement tends to be saturated, which may be affected by redundant parameters. The MMCD model proposed in this paper makes structural innovations in the multi-modal fusion strategy, which not only introduces a cross-modal attention mechanism and an efficient convolution module, but also optimizes the interaction path of image–text information. MMCD achieves the highest Accuracy 97.96%, Precision 98.13%, Recall 97.96%, and F1 score 97.97% while maintaining a moderate parameter scale (64.25 M) and a relatively low computational cost (3.80 GFLOPs).

As can be seen from Table 5, the Precision and Recall of the basic image model ResNet50 in the Health category are only 75.26% and 89.26%, respectively, indicating that it has obvious misdetection problems and insufficient ability to model the boundary between healthy feces and early mild feces. At the same time, the F1 value of ResNet50 in the Ncd category is only 83.23%, which may be affected by feature confusion. The improved ResNet50-MD model improves in all categories, especially in the Salmo category, where the F1 reaches 90.84%, which verifies the important role of the multi-scale strategy for the detail recognition of stool images.

**Figure 10:**
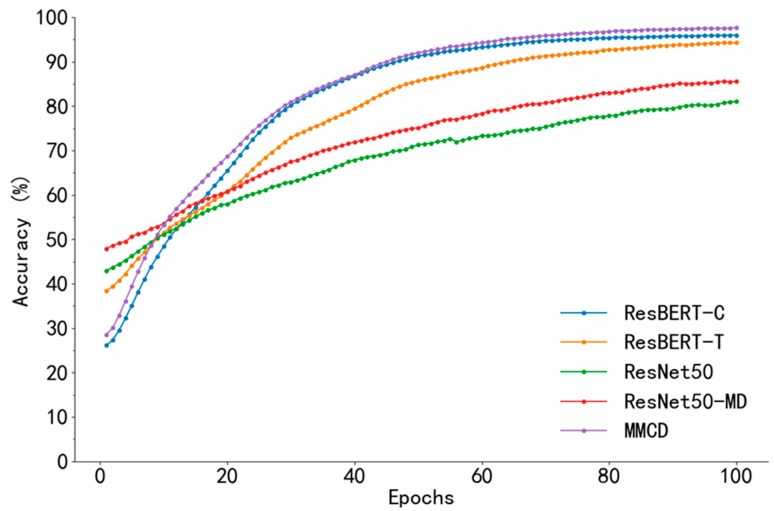
Comparison of test accuracy across different fusion methods.

The overall performance of the multimodal fusion model in each category tended to be consistent, and the F1 values of the four categories were all above 90%, especially in Cocci and Salmo categories (F1 of Salmo category was 96.98% and 97.90%, respectively). This indicates that semantic description can assist in image discrimination, especially in diseases with small differences in appearance but significant differences in semantics, and text information provides a valuable basis for discrimination. The Precision and Recall of the MMCD model proposed in this paper reach 98% or more in the four categories, and the F1 score of the Ncd category reaches 95.74%. In addition, the Recall of the Health category is as high as 96.85%, which is significantly better than that of other models. As can be seen from Figure 10, the Accuracy of the proposed model MMCD on the test set is significantly improved compared with the single-modal model and the models using other fusion methods.

The above experiments show that the proposed model MMCD has stronger fine-grained identification ability and generalization ability, and has more advantages in dealing with fuzzy boundaries between healthy and diseased feces. Moreover, MMCD achieves a better trade-off among accuracy, computational efficiency, and model complexity, which shows strong potential for practical application.

### 3.3. Ablation Experiments

#### 3.3.1. GCA Activation Function and Cross-Attention Head Number Ablation Experiments

To validate the impact of different activation functions and cross-attention head counts on model classification accuracy, this section designs ablation experiments targeting the internal activation functions and cross-attention head counts of GCA. The activation functions selected were Sigmoid, Tanh, and ReLU, while the number of attention heads was set to 4, 8, and 12, covering a range from “computational efficiency” to “fine-grained feature capture,” aligning with GCA’s requirements for cross-modal association modeling.

As shown in Table 6, the accuracy of the Sigmoid + 8 combination (97.96%) is significantly higher than that of Tanh + 8 (95.82%) and ReLU + 8 (94.99%), indicating that its “noise suppression” characteristic is more suitable for multimodal fecal detection tasks. In fecal detection, the association between textual descriptions (e.g., “dark brown and viscous”) and image features remains relatively stable. Sigmoid suppresses irrelevant modalities (e.g., bedding noise), enabling it to focus more precisely on effective features. All activation functions reach their performance peak at eight heads, and accuracy decreases beyond eight heads (e.g., Sigmoid from 97.96% to 96.95%). Excessive head counts can lead to attention dispersion and increased interference between modalities (weak associations between text and images are incorrectly amplified), while 8 heads strike a balance between “modality correlation granularity” and “computational efficiency.” Tanh’s bidirectional regulation capability (which can enhance effective features) is theoretically more suitable for multimodal tasks, but its performance is inferior to Sigmoid in experiments, as “suppressing noise” takes priority over “enhancing features” in low-quality fecal classification. ReLU’s non-negative activation causes a loss of negative input information, performing worst in scenarios with significant modal fluctuations (e.g., low-quality images) (ReLU + 8 accuracy rate is only 94.99%).

As shown in Table 7, in the Health category, most combinations demonstrated high performance (e.g., Sigmoid + 4 with Precision: 99.52%, Recall: 91.96%, F1 score: 95.59%). However, the Sigmoid + 8 combination further optimizes semantic feature extraction and multimodal information fusion through the synergistic effect of the Sigmoid activation function and 8-head cross-attention, ultimately achieving the optimal performance of 100% Precision, Recall, and F1 score, indicating that this combination has extremely strong stability and accuracy in identifying health samples. The Cocci category exhibits significant shortcomings in certain combinations, such as Tanh + 4, which has a Recall of only 86.86% and an F1 score as low as 92.07%, reflecting a severe missed detection of cocci infection samples by the model. The Sigmoid series combinations perform better in this category, with Sigmoid + 8 maintaining 100% Precision while increasing Recall to 92.70% and achieving an F1 score of 96.21%, significantly reducing the risk of missed detection and effectively balancing identification accuracy and coverage for this category of samples. For the Ncd category, some combinations exhibit imbalances between Precision and Recall rates. For example, ReLU + 4 has a Recall rate of only 88.58% despite a high Precision rate of 94.94%, but its F1 score is only 91.65%, indicating insufficient identification stability. The Sigmoid + 8 combination achieves 100% Recall in this category, while improving Precision to 91.84% and achieving an F1 score of 95.74%, ensuring comprehensive capture of Newcastle disease samples while significantly reducing misclassifications, thereby achieving more reliable identification results. The Salmo category already exhibits good performance in most combinations, but the Sigmoid + 8 combination still demonstrates a significant advantage. In contrast, the Precision of ReLU + 4 is only 89.32%, and while Tanh + 4 has a high Recall (99.61%), its Precision is relatively low (91.49%). Meanwhile, Sigmoid + 8 achieves a perfect 100% in Precision, Recall, and F1 score for this category, fully validating its powerful capability in distinguishing subtle pathological features such as Salmonella infection.

Overall, the Sigmoid + 8 combination achieved optimal or near-optimal performance across all four categories, particularly achieving “zero misclassifications and zero false negatives” in the Health and Salmo categories while effectively balancing recognition accuracy and coverage in the Cocci and Ncd categories. With an overall Accuracy of 97.96%, it emerged as the best choice among all combinations, fully demonstrating the synergistic advantages of the Sigmoid activation function and 8-head cross-attention in the task of multi-modal classification of sick chicken feces.

#### 3.3.2. Ablation Experiments for Each MMCD Module

In order to thoroughly assess the contributions and synergistic effects of each component within the MMCD model, this study implemented a systematic ablation experiment. The process commenced with a basic Concat concatenation model, referred to as ResBERT-C. Subsequently, the DSconv module, the Manhattan attention mechanism (MASA), and the Gated Cross-Attention fusion module (GCA) were sequentially incorporated. This iterative integration culminated in the development of a comprehensive MMCD bimodal fusion model, which facilitated the analysis of the impact of each module on the model’s performance. The results of these experiments are presented in Table 8 and Table 9.

It can be seen from Table 8 that the base model ResBERT-C has the weakest performance in various performance indicators, indicating that the model is still unable to fully extract and fuse the information of image and text modalities. After the introduction of DSconv, the F1 value of DSconv was increased by 1.37 percentage points under the premise of a 4.2% reduction in the number of parameters (58.18 M vs. 60.71 M) and a similar computational cost (3.68 G vs. 3.67 G). This shows that the Depthwise Separable convolution effectively improves the efficiency of feature representation by focusing on the key feature regions, and it verifies the necessity of the local feature screening mechanism. After adding the MASA module (ResBERT + MASA), although the parameters increased by 16.5% (70.74 M vs. 60.71 M), the F1 is improved to 97.04% and the Accuracy was 97.05%, which verifies the effectiveness of MASA in feature recollection and that it can effectively capture the cross-level semantic association. With the introduction of a GCA module (ResBERT + GCA) at a similar parameter scale (71.75 M vs. 70.74 M), GCA obtains better parameter efficiency than MASA and improves the F1 to 96.94%. After further combining two or more modules, the performance continues to improve. For example, the Accuracy of the ResBERT + MASA + GCA model is 96.95%, which is almost optimal. The MMCD model, with a complete fusion of three modules, achieves the best performance on all indicators, its parameter number is only 64.25 M, and the calculation amount is 3.80G, showing a superior balance between accuracy and computational efficiency.

As can be seen from Table 9, in the Health category, the baseline model showed high performance (Precision: 99.10%, Recall: 98.66%, F1 score: 98.88%). However, by introducing the MASA module, it was further optimized in terms of semantic consistency and spatial attention, and it finally achieved the optimal performance of 100% in Precision, Recall, and F1 value in the MMCD model, indicating that the framework has strong generalization ability in identifying health samples. Under the baseline model, the Recall rate of the Cocci category was only 84.67%, and the F1 value was also significantly low, which reflected the missed detection phenomenon of the model. After the introduction of the DSconv and MASA modules, the Recall rate stably increased to more than 91%, and the F1 value increased synchronously, which significantly reduced the risk of missed detection. Finally, the MMCD model increased the Recall rate to 92.70% while maintaining 100% Accuracy, which effectively balanced the recognition ability and the risk of misjudgment. For the Ncd category, although the Recall rate of the baseline model reached 100%, its Precision was only 88.24%, indicating that there was a large number of misjudgments. After the introduction of the MASA module, the discrimination ability of the model for this category of samples was significantly enhanced, and the Accuracy and F1 value were improved. The MMCD model improves the Accuracy to 91.84% and the F1 value to 95.74% while maintaining a high Recall rate, realizing a more stable and accurate recognition of this type of disease. The Salmo category had high performance under the baseline model, but there was still room for improvement through module-level enhancement. After the introduction of MASA and GCA, the Precision and Recall of the Salmo category were improved. Finally, the MMCD model achieved 100% performance in Precision, Recall, and F1 score in this category, which fully verifies its advantages in dealing with subtle pathological differences.

In summary, the ablation experiments clearly reveal the functional positioning of each component module in the MMCD model and their contribution to the overall performance, and the performance improvement of each module on different categories is different. The combination of MASA and GCA has significant synergistic advantages in multimodal scenarios. Although DSconv can improve the convolution efficiency, it needs to be carefully introduced in the fusion structure to avoid redundancy. The MMCD model constructed by the organic combination of DSconv, MASA, and GCA shows stable and excellent classification performance in all categories. This indicates that the synergy of multi-level semantic alignment and multi-modal feature fusion is a key path to achieving high-precision avian disease diagnosis. The complete MMCD model shows robustness and high accuracy in multi-class recognition, which verifies technical effectiveness of the multi-modal collaborative design strategy in this paper.

**Figure 11:**
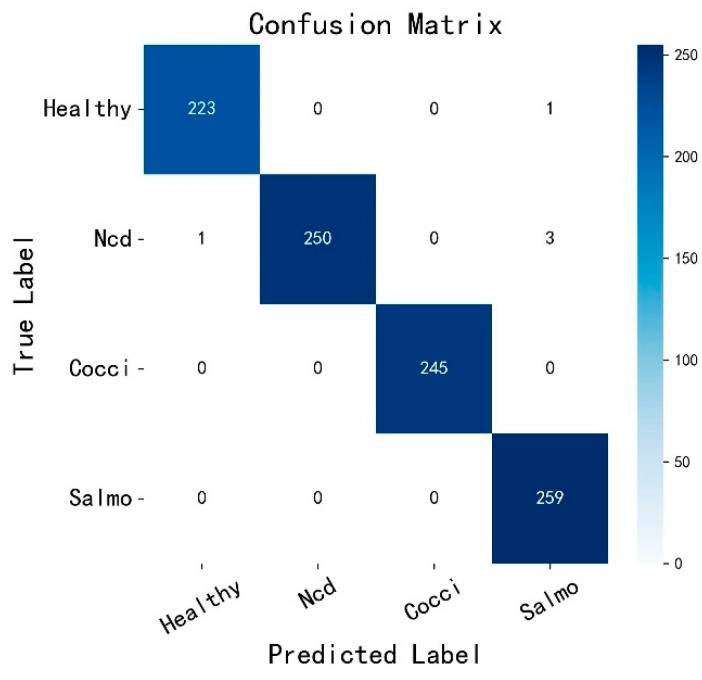
MMCD confusion matrix.

As shown in Figure 11, the model’s misclassifications occurred in the Health and Ncd categories, while all other categories are correctly classified. By analyzing the image information and text descriptions of the misclassified images in Table 10, it was found that the misclassifications were due to the similarity of features between the two categories in some samples, which are prone to misjudgment under low-light or blurry conditions. Additionally, the boundary features in Ncd images are less distinct compared to other categories, and Health samples exhibit a “long, narrow shape,” while some Ncd samples are “irregularly clustered.” When the shape of Ncd feces resembles a long, narrow shape, the boundary between it and the Health category becomes blurred. Keywords such as “white components” and “green” appear in both categories. If image features are unclear, the discriminative power of textual semantics is weakened. However, overall, the dual-modal fusion model for detecting pathological chicken feces still achieves a significant improvement in classification accuracy compared to single-modal image classification.

The experimental findings presented above indicate that the integration of a Gated Cross-Attention (GCA) module is effective in fusing image and text modalities, thereby enhancing stability and generalization capabilities. In contrast to simple concatenation, the gating mechanism allows for the dynamic adjustment of weights assigned to different modal information, which enables the model to more flexibly leverage pertinent information during classification tasks. Concurrently, the cross-attention mechanism adeptly captures semantic associations between image and text modalities, resulting in a notable improvement in classification accuracy. Nonetheless, despite the observed enhancements across various metrics with cross-modal Transformer encoders, the increased number of layers contributes to heightened computational complexity, which can lead to suboptimal performance in certain categories. This shows that a balance between model complexity and performance needs to be found in multimodal tasks.

## 4. Discussion

In this study, the three major challenges in poultry pathology fecal detection consisting of weak feature expression, modal limitations, and environmental interference (as described in the Introduction) were effectively addressed by constructing a multimodal fusion model, MMCD. Experiments have demonstrated that the bimodal strategy of fusing visual and textual semantics significantly improves the discriminative ability of pathology features. The core value of this study is reflected in three aspects: first, technological innovation and performance breakthrough. Integrating the MASA mechanism with DSconv in the ResNet50 backbone network successfully mitigates the feature confusion problem caused by the high visual similarity of feces. The amount of model parameters is reduced by 7.5 M, and the computation amount is reduced by 1.62 G, providing a lightweight foundation for agricultural edge computing scenarios. Migration extraction of textual semantic features (e.g., breeding records, symptom descriptions) using a pre-trained BERT compensates for the lack of characterization of weak pathological features by a single image modality. This method reduces the dependence on manual annotation and solves the problem of “fuzzy boundary of latent features” pointed out in the introduction. The proposed Gated Cross-Attention (GCA) module effectively suppresses environmental noise such as matting color/lighting unevenness by using a dynamic weighted screening of key pathological features, reduces the number of parameters by 41% compared with the cross-modal Transformer, breaks through the bottleneck of redundancy of information in the existing methods [17,18,19,20,21,22,23], and improves the classification Accuracy of Concat by 2.51–2.82%, which demonstrates the superiority and ability of adaptive fusion. Second, performance: Compared with the traditional unimodal methods, the model in this study has advantages in Accuracy (+8.69%), Recall (+8.72%), Precision (+8.67%) and F1 value (+8.72%), which confirms the ability of multimodal fusion to crack the “high visual similarity of fecal matter of multiple etiologies” (as described in the Abstract); furthermore, through the image degradation enhancement strategy, the model maintains stable performance in complex aquaculture environments, which directly addresses the challenge of “environmental disturbances reduce model robustness” emphasized in the introduction. Third are the contributions to the field and paradigm innovation. For the first time, the image–text fusion paradigm is introduced into the field of livestock and poultry pathology detection, which extends the application boundary of multimodal learning in agricultural health monitoring. Compared with existing disease studies [17,18,19,22], this study verifies the enhancement effect of text modality on animal micropathology features. By improving the timeliness and accuracy of fecal pathology identification (the core objective of the abstract), we can provide technical support to reduce antibiotic misuse, and early and accurate diagnosis can reduce the reliance on drugs for prevention and control of more than 80 laying hens’ diseases [3], directly addressing the problem of “health risks caused by drug residues”, as emphasized in the introduction. The lightweight design of the GCA gives the model potential to be deployed on the edge of the farm, which can promote the intelligent monitoring system to move from “sensing” to “decision making”.

Despite the excellent performance of MMCD, there are still challenges for scale-up applications. First, the text modality relies on expert descriptions, and semantic subjectivity may lead to fluctuations in model discrimination. In the future, automatic caption generation [32] based on pre-trained visual language models (e.g., BLIP [33]) can be explored, and the conversion of fecal images to standardized text can be achieved through model fine-tuning to reduce the reliance on expert descriptions and eliminate manual description bias; second, in complex farming scenarios, the model computational efficiency (64.25 M/3.80 G) still struggles to meet the real-time deployment requirements of low-power devices, and the model computation efficiency needs to be further optimized to adapt to the low-latency deployment of edge devices. In the future, a cross-modal knowledge distillation framework [34] can be designed to compress the bimodal knowledge into a lightweight network (e.g., MobileNetV3 [35]) to improve the inference speed at the edge; third, feature similarities between different categories of features in some samples are easily misjudged in low-light or blurred images ((e.g., “healthy” and “n”) and “ncd”), which elevates the risk of misdetection. In the future, we can try to introduce the introduction of depth sensors [36] to construct a RGB-Depth-Text multimodal system and utilize depth information to resolve the geometric features (e.g., feces height/density) of overlapping targets in 2D vision, so as to solve the problem of interclass confusion.

## 5. Conclusions

The health status of chickens can be evaluated through an analysis of fecal characteristics. Chickens in poor health often display notable deviations in fecal color, shape, and texture, including darkened coloration, a thin consistency, or the presence of atypical components. Nonetheless, reliance solely on visual assessment may not provide a comprehensive reflection of the chickens’ health status, particularly when features are subtle or overlapping, potentially resulting in inaccurate diagnostic outcomes. Furthermore, in practical farming settings, variables such as the performance of data collection devices, lighting conditions, and surface reflections on feces can contribute to image data that are characterized by low resolution, elevated noise levels, and blurred focus.

In order to solve the above problems, this paper constructs a low-quality dataset under different illumination and noise conditions and proposes an innovative diagnosis model for sick chicken feces. The model fuses image and text information. Compared with the use of computer vision technology to extract feces from images for classification and recognition, the accuracy is greatly improved, and the tedious process of data labeling is significantly simplified. Specifically, this paper uses the improved ResNet network to extract stool image features, combines with the BERT model to extract text description features, and realizes the efficient integration of information through the multimodal fusion method GCA, so as to reduce the cost required for manually cropping images and labeling during training while ensuring good diagnostic performance.

The experimental results show that compared with the single-modal model, the proposed dual-modal fusion model (MMCD) shows a significant improvement in diagnostic performance, especially in evaluation indicators such as Accuracy, Recall rate, and F1 score. Although the cross-modal Transformer encoder also plays a positive role in feature fusion, its comprehensive performance fails to surpass the GCA fusion method due to its high computational complexity. The Gated Cross-Attention (GCA) method was combined to further optimize the information interaction process between different modalities, thereby significantly improving the stability and generalization ability of the model. The Accuracy of the improved dual-modal fusion model for the diagnosis of sick chicken feces reached 97.96%, which was 11.61%, 12.38%, 12.24%, and 12.38% higher than that of the basic ResNet50 in each index. Compared with the improved ResNet50, the Accuracy of the proposed model was increased by 8.69%, 8.72%, 8.67% and 8.72% in each index. Compared with simple Concat concatenation, each index was increased by 2.51%, 2.75%, 2.82% and 2.75%. Compared with the cross-modal Transformer encoder, each index was increased by 2.69%, 2.85%, 2.87%and 2.85%. Compared with the basic ResNet50, the MMCD model using the improved ResNet50 had parameters reduced by 7.5M, and the calculation amount reduced by 1.62G.

## Figures and Tables

**Table 1:** Partial data examples.

Image	Text	Class	Label
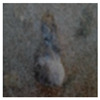	The chicken manure, which is distributed in the center of the image, is dark green with some white and has a long strip shape	healthy	0
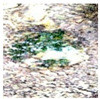	Chicken manure appears emerald green and is distributed in the center of the image, with parts of white uric acid on the surface and irregular clumps in shape	ncd	1
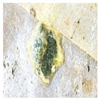	Chicken manure presents a pool shape, distributed on the ground, with chicken manure in the middle being green, and the surrounding light yellow, extremely thin	ncd	1
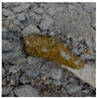	It is distributed on the stone pile, showing a dark brown, relatively sticky texture, and the shape tends to be long strips	cocci	2
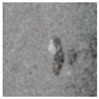	Chicken dung is dark brown, with some white above, long strips, and dry and hard in texture	salmo	3

**Table 2:** Number of dataset samples.

Categories	Training Set	Validation Set	Test Set	Totals
Healthy	1701	479	224	2404
Ncd	1602	451	254	2307
Cocci	1749	482	245	2476
Salmo	1816	550	259	2625
Total	6868	1962	982	9812

**Table 3:** Hardware configuration and experimental environment.

Hardware	Configuration	Environment	Versions
Operating system	Windows10	Python	3.8
CPU	Intel(R) Core(TM) i7-9700	PyTorch	2.2.1
GPU	NVIDIA GeForce RTX 3060	Cuda	12.1

**Table 4:** Comparison between single-modal and multi-modal fusion.

Models	Precision (%)	Recall (%)	F1 (%)	Accuracy (%)	Parameters (M)	Flops (G)
ResNet50	86.52	85.58	85.73	85.58	25.56	4.13
ResNet50-MD	89.44	89.24	89.30	89.24	18.06	2.51
ResBERT-C	95.62	95.21	95.15	95.21	60.71	3.78
ResBERT-T	95.44	95.11	95.10	95.11	145.79	4.86
MMCD	98.13	97.96	97.97	97.96	64.25	3.80

**Table 5:** Comparison of each category between single-modal and dual-modal fusion.

Model	Precision (%)				Recall (%)				F1 (%)				Accuracy (%)
	Health	Cocci	Ncd	Salmo	Health	Cocci	Ncd	Salmo	Health	Cocci	Ncd	Salmo	
ResNet50	75.26	94.04	82.01	93.75	89.26	89.11	84.48	79.85	81.66	91.51	83.23	86.24	85.58
ResNet50-MD	84.06	96.55	85.48	91.19	87.19	90.32	88.79	90.49	85.60	93.33	87.10	90.84	89.24
ResBERT-C	99.10	99.57	88.24	94.83	98.66	84.67	100	99.23	98.88	91.52	93.75	96.98	95.21
ResBERT-T	97.73	98.77	87.75	96.62	95.98	87.59	98.67	99.23	96.85	92.84	92.89	97.90	95.11
MMCD	100	100	91.84	100	100	92.70	100	100	100	96.21	95.74	100	97.96

**Table 6:** GCA internal component ablation experiment.

Model	Precision (%)	Recall (%)	F1 (%)	Accuracy (%)
Sigmoid + 4	95.33	95.11	95.11	95.11
Sigmoid + 8	98.13	97.96	97.97	97.96
Sigmoid + 12	97.11	96.95	96.95	96.95
Tanh + 4	95.00	94.70	94.68	94.70
Tanh + 8	96.03	95.82	95.81	95.82
Tanh + 12	97.02	96.84	96.84	96.88
ReLU + 4	93.66	93.48	93.48	93.55
ReLU + 8	95.41	95.11	95.08	94.99
ReLU + 12	94.18	93.97	93.99	93.97

**Table 7:** GCA internal component ablation experiment for each category.

Model	Precision (%)				Recall (%)				F1 (%)				Accuracy (%)
	Health	Cocci	Ncd	Salmo	Health	Cocci	Ncd	Salmo	Health	Cocci	Ncd	Salmo	
Sigmoid + 4	99.52	97.67	91.25	92.78	91.96	91.97	97.33	99.23	95.59	94.74	94.19	95.90	95.11
Sigmoid + 8	100	100	91.84	100	100	92.70	100	100	100	96.21	95.74	100	97.96
Sigmoid + 12	100	98.44	91.46	98.09	96.88	92.34	100	99.23	98.41	95.29	95.54	98.66	96.95
Tanh + 4	100	97.94	90.50	91.49	95.98	86.86	97.33	99.61	97.95	92.07	93.79	95.38	94.70
Tanh + 8	93.31	99.60	91.60	98.45	99.55	89.78	96.89	98.07	96.33	94.43	94.17	98.26	95.82
Tanh + 12	100	98.82	91.46	97.35	97.32	91.61	100	99.23	98.64	95.08	95.54	98.28	96.88
ReLU + 4	92.64	97.85	94.94	89.32	95.54	93.06	88.58	96.91	94.07	95.40	91.65	92.96	93.55
ReLU + 8	97.31	100	91.80	92.03	96.88	87.23	99.56	98.07	97.09	93.18	95.52	94.95	94.99
ReLU + 12	89.41	99.55	94.78	92.96	95.00	90.28	94.78	95.80	92.12	94.69	94.78	94.36	93.97

**Table 8:** Ablation experiment results.

Model	Precision (%)	Recall (%)	F1 (%)	Accuracy (%)	Parameters (M)	Flops (G)
ResBERT-C	95.62	95.21	95.15	95.21	60.71	3.67
ResBERT + DSconv	96.73	96.54	96.52	96.54	58.18	3.68
ResBERT + MASA	97.18	97.05	97.04	97.05	70.74	5.40
ResBERT + GCA	97.09	96.95	96.94	96.95	71.75	5.31
ResBERT + DSconv + MASA	97.02	96.84	96.82	96.84	68.21	5.31
ResBERT + DSconv + GCA	96.57	96.44	96.42	96.44	61.72	3.69
ResBERT + MASA + GCA	97.09	96.95	96.94	96.95	74.28	5.41
MMCD	98.13	97.96	97.97	97.96	64.25	3.80

**Table 9:** Class-wise results of ablation experiments.

Model	Precision (%)				Recall (%)				F1 (%)				Accuracy (%)
	Health	Cocci	Ncd	Salmo	Health	Cocci	Ncd	Salmo	Health	Cocci	Ncd	Salmo	
ResBERT-C	99.10	99.57	88.24	94.83	98.66	84.67	100	99.23	98.88	91.52	93.75	96.98	95.21
ResBERT + DSconv	98.67	99.60	91.46	96.59	99.11	89.78	96.52	100	98.89	94.43	95.54	97.51	96.54
ResBERT + MASA	99.55	98.05	91.84	98.83	99.55	91.97	100	97.68	99.55	94.92	95.74	98.25	97.05
ResBERT + GCA	99.11	99.21	91.77	97.72	99.11	91.24	99.11	99.23	99.11	95.06	95.30	98.47	96.95
ResBERT + DSconv + MASA	99.12	99.60	91.84	96.97	100	89.78	100	98.84	99.56	94.43	95.74	97.90	96.84
ResBERT + DSconv + GCA	96.55	98.03	91.77	99.21	100	90.88	99.11	96.91	98.25	94.32	95.30	98.05	96.44
ResBERT + MASA + GCA	99.11	99.21	91.77	97.72	99.11	91.24	99.11	99.23	99.11	95.06	95.30	98.47	96.95
MMCD	100	100	91.84	100	100	92.70	100	100	100	96.21	95.74	100	97.96

**Table 10:** Typical examples of misclassified images.

Image	Text	Class	Label
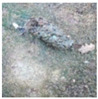	Chicken manure is distributed in the center of the image, with a dark brown color and a long, strip-like shape, and the surface has some white uric acid.	healthy	0
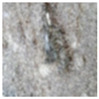	Chicken manure is dark brown in color, relatively moist, with white substances on the surface, and appears in long strips.	ncd	1

## Data Availability

The original contributions presented in this study are included in the article. Further inquiries can be directed to the corresponding author(s).
